# 经支气管针吸活检在肺部疾病诊治中的作用

**DOI:** 10.3779/j.issn.1009-3419.2010.05.03

**Published:** 2010-05-20

**Affiliations:** 100730 北京，中国医学科学院中国协和医科大学北京协和医院呼吸内科 Department of Respiratory Medicine, Peking Union Medical College Hospital, Chinese Academy of Medical Sciences and Peking Union Medical College, Beijing 100730, China

**Keywords:** 经支气管针吸活检, 经支气管超声引导针吸活检, 肺肿瘤, 诊断, 分期, TBNA, EBUS-TBNA, Lung neoplasms, Diagnosis, Staging

## Abstract

经支气管针吸活检（transbronchial needle aspiration, TBNA）在其临床应用的三十余年里，以其高效、准确、创伤小等特点得到了临床医师们的青睐。支气管内超声（endobronchial ultrasound, EBUS）的应用更增加了TBNA的准确性。EBUS-TBNA更比TBNA和其它传统的检查方法（如CT、PET、纵隔镜等）的敏感性、特异性和准确性高并有较高的安全性。TBNA可获得组织学标本，对于肺癌的诊断和分期有重要意义。同时对肺部其它疾病（如结节病、纵隔淋巴瘤、肺结核）的诊断，TBNA也有帮助。TBNA联合应用常规检查方法可提高肺部疾病的诊断率和阴性预测值，在临床工作中应大力推广TBNA的使用。

## Abstract (en)

In the last 30 years, clinical application of transbronchial needle aspiration (TBNA) has proved its efficiency, accuracy, safety and cost-effectiveness, particularly in diagnosing and staging lung cancer as well as in diagnosing benign diseases. The application of endobronchial ultrasound (EBUS) increased the accuracy of TBNA. Endobronchial ultrasound guided transbronchial needle aspiration (EBUS-TBNA) has superior performance and allowing real-time sampling under direct vision. Combination of EUBS-TBNA and traditional methods (CT, PET, mediastinoscopy, *et al*) can improve the diagnostic rate and negative predictive value. In the future, EUBS-TBNA may have applications in both benign and malignant lung diseases as a routine examination.

可曲支气管镜检查是呼吸科常用的检查方法之一，观察到可疑病灶即行活检和毛刷，但对于纵隔肺门淋巴结肿大或粘膜下病变等情况，常规方法不能明确诊断。20世纪80年代，经支气管针吸活检（transbronchial needle aspiration, TBNA）开始应用于临床，利用该技术可以获得纵隔肺门淋巴结的细胞学和/或组织学标本，从而明确诊断。TBNA虽然是一种有创检查，但是其损伤小，安全性很高，感染性并发症很少见^[1, 2]^。本文对其临床应用和进展进行综述。

## 1 TBNA的发展

TBNA是指利用特制的带有可弯曲导管的穿刺针，通过支气管镜的活检孔道进入气道，穿透气管支气管壁进入病灶内，负压吸引获得细胞学和/或组织学标本。1949年TBNA之父阿根廷Schieppati开展硬质气管镜下隆突下淋巴结穿刺；1983年美国Wang开始应用纤维支气管镜，通过改进穿刺针和总结穿刺方法提高了诊断准确性；于2000年日本开始应用凸面支气管内镜超声引导TBNA（convex probe endobronchial ultrasound TBNA, CPEBUS-TBNA），进一步提高了TBNA的准确性。

Herth等^[3]^比较了常规TBNA和先行EBUS然后进行TBNA的诊断效果，结果显示穿刺成功（穿刺物可见淋巴组织或得到明确诊断）的比例对于隆突下淋巴结（共100例）分别为74%和86%；对于其它部位淋巴结（共100例）分别为58%和84%，可见EBUS的主要作用是非隆突下淋巴结的定位。Kanoh等^[4]^比较了实时EBUS-TBNA与先行EBUS然后进行TBNA的诊断效果，结果显示实时EBUSTBNA的准确性为97%，而先行EBUS然后进行TBNA的准确性为76%。因此Olymous公司发明了CP-EBUS-TBNA，是一种真正的实时EBUS-TBNA。Yasufuku等^[5]^采用新设备穿刺70例疑似肺癌的患者，其中58例纵隔淋巴结和12例肺门淋巴结，结果显示其敏感性为95.7%、特异性为100%。Vilman等对573枚淋巴结的总结显示其总敏感性为92%；特异性为100%；准确性为94%。

## 2 TBNA对肺癌的诊断

TBNA有助于肺癌的确诊，是取得肺癌病理学结果的有效方法之一，而且创伤性小、安全性好。Stratakos等^[6]^用19 G穿刺针诊断肺癌淋巴结转移，共77例患者接受了检查，其中66例有肺癌淋巴结转移，TBNA诊断的敏感性为87.9%（58/66），其中小细胞肺癌为94%。王孟昭等^[7]^分析了北京协和医院2年间77例接受TBNA的患者，其中38例诊断为肺癌。77例患者共穿刺128个部位，225针，其中TBNA穿刺成功222针（98.7%）。38例肺癌患者中，TBNA总结果阳性31例（81.6%），其中9例患者TBNA涂片为唯一病理学证据。38例肺癌患者共穿刺63枚淋巴结，其中TBNA结果阳性41例（65.1%）。TBNA结果的阳性率与病理类型和淋巴结大小有关。13项TBNA试验荟萃分析，每项入组10例-183例患者，其中6项试验有手术病理证实，平均纵隔淋巴结转移率为34%，TBNA总的敏感性和特异性分别为39%和99%；7项试验无手术病理证实，平均纵隔淋巴结转移率为81%，TBNA总的敏感性为78%。13项试验共报道了2例大出血、1例气胸需要胸腔引流、2例气胸和1例纵隔气肿不需要引流，总的并发症为0.26%。

Patel等^[8]^回顾性分析了本单位8年的资料，根据TBNA的方法分为早期组（采用19 G或21 G穿刺针仅获得细胞学标本）和后期组（采用19 G活检针获得组织学标本）。结果显示两种方法获得满意标本的比例分别为53%和91%，避免手术活检的比例分别为35%和66%，两组均有显著差别。Skov等^[9]^分析了内镜超声引导针吸活检（EUS guided fine needle aspiration, EUS-FNA）和TBNA涂片病理学检查的可重复性和影响因素。共102张涂片，由4位病理学医师进行检查2次，2次之间接受简单培训。4位病理学医师均有15年病理学检查经验，但检查EUS-FNA和TBNA涂片的经验相差很大，有些没有，有些有10年经验。结果显示第一次检查的可重复性很好，但通过培训，重复性可进一步提高。因此可见内镜医师的穿刺技术和病理学医师的检查技术均可以通过培训和经验的积累而提高。

通过传统支气管镜很难取得位于中央型肺实质病变组织，因此对中央型肺癌诊断的特异性不高。对于非诊断性显微支气管镜疑似肺癌的患者，可通过EBUS-TBNA进一步检查确认。Tournoy等^[10]^回顾性分析了60例疑似肺癌的中央型肺实质病变患者。这些患者接受EBUS-TBNA，如果EBUS-TBNA不能得出恶性疾病的病理诊断，则通过经胸穿刺活检或手术来确诊。82%的患者之前做过非诊断性纤维支气管镜。所有的病例在超声下均可见原发性肺病变。其中46例（77%）可通过EBUS-TBNA确诊为肺癌，敏感性为82%（95%CI: 69%-91%），阴性预测值为23%（95%CI: 5%-53%）。通过EBUS-TBNA，可使患者行经胸穿刺活检或手术诊断的人数分别减少47%和30%。由此可见，EBUSTBNA可作为中央型肺实质病变患者行非诊断性支气管镜后的诊断性检测。虽然EBUS-TBNA的诊断率和敏感性较高，但是阴性预测值却并不令人满意^[11, 12]^。原因是一些小的转移灶不易被EBUS-TBNA获取。因此对于EBUS-TBNA阴性但其它检查高度怀疑肺癌或生活于癌症高发区的患者应对阴性淋巴结进行活检。

Herth等^[13]^近期进行了一项在单一支气管镜下联合应用EBUS-TBNA和EUS-FNA的研究。通过联合应用EBUSTBNA和EUS-FNA，在150例疑似非小细胞肺癌（non-small cell lung cancer, NSCLC）的病例中，139例可以被确诊（其中83例为男性，占91%；56例为女性；平均年龄为57.6岁）。对EBUS-TBNA和EUS-FNA检查为阴性的病例，通过手术或临床随访来确定。在被确诊的139例患者中，共有619枚淋巴结被穿刺活检。其中EUS-FNA检测了229枚，EBUSTBNA检测了390枚，敏感性分别为89%和92%。而联合应用的敏感性为96%，阴性预测值为95%，均优于单用任何一种检测方法。

穿刺所得到的组织除进行病理学检查外，还可进行其它检查。Nakajima等^[14]^利用EBUS-TBNA的组织进行*EGFR*基因突变检测，对46例患者的石蜡包埋的EBUSTBNA的组织进行分析。结果显示43例可进行分析，其中11例（25.6%）有外显子19/21突变。近期Garcia-Olivé等^[15]^对20例有淋巴结转移的肺腺癌患者进行了EBUS-TBNA。对穿刺组织提取DNA进行*EGFR*基因突变检测，其中2例（10%）有外显子19/21突变。

## 3 TBNA在肺癌分期中的作用

纵隔肺门淋巴结的转移情况对于NSCLC的分期非常重要，并决定治疗方案和治疗效果。因此取得纵隔肺门淋巴结细胞学和/或病理学样本，对NSCLC有着重要意义。TBNA技术用于肺癌的分期要求能准确进行较小淋巴结的穿刺。由于纵隔镜不能穿刺肺门淋巴结，因此EBUS-TBNA在肺门淋巴结分期中的作用更为显著。Yasufuku等^[16]^比较了CT、PET和EBUS-TBNA在肺癌患者纵隔淋巴结诊断中的作用。共102例患者，包括147枚纵隔淋巴结和53枚肺门淋巴结。EBUS-TBNA证实24例患者中37站淋巴结阳性。CT、PET和EBUS-TBNA诊断纵隔和肺门淋巴结转移的敏感性分别为76.9%、80.0%和92.3%；特异性分别为55.3%、70.1%和100%，准确性分别为60.8%、72.5%和98.0%。TBNA的敏感性和特异性均明显高于CT和PET。另一项多中心研究^[17]^对213例PET或CT肺门淋巴结阳性的患者进行了EBUS-TBNA。之后通过外科手术分级和临床随访作为标准。研究显示EBUSTBNA的敏感性、特异性和阳性诊断率分别为91%、100%和92.4%。因此对CT/PET阳性的疑似肺癌患者进行EBUSTBNA可提高诊断率，并对准确分期有帮助。

EBUS-TBNA对不同组织类型的NSCLC纵隔淋巴结的诊断率不同。Hwangbo等^[18]^对EBUS-TBNA和CT/PET结果进行了亚组分析。对于腺癌患者，EBUS-TBNA的阴性预测值高于CT/PET（94.6% *vs* 77.8%, *P*=0.044）；而对于鳞癌患者，两者的假阴性率相似。因此，EBUS-TBNA对于腺癌患者的纵隔淋巴结分级更有意义。

对于直径小于1 cm的纵隔淋巴结，TBNA有无意义呢？Herth等^[19]^总结了100例NSCLC患者，共119枚淋巴结，直径5 mm-10 mm。通过手术证实，EBUS-TBNA诊断了19例患者有纵隔淋巴结转移，但2例假阴性。诊断的敏感性、特异性和准确性分别为92.3%、100%和96.3%。Hsu等^[20]^研究了常规TBNA对小纵隔淋巴结的诊断价值，回顾性分析了19例患者，PET/CT共发现了25枚可疑淋巴结，淋巴结短轴直径平均为9.9 mm±3.0 mm，接受常规TBNA检查。结果显示敏感性、特异性和准确性分别为81.8%、100%和84%，每枚淋巴结平均穿刺（2.36±0.49）次。25枚淋巴结中有9枚（36%）小于1.0 cm，准确性和敏感性分别为88.9%和87.5%。证实了对于小的肺门纵隔淋巴结，TBNA也可获得病理学结果，也有很大地诊断意义。

Felix等^[21]^对CT和PET纵隔淋巴结阴性的临床Ⅰ期NSCLC患者进行了EBUS-TBNA，并经手术验证。156枚直径5 mm-10 mm的淋巴结被检测，其中手术证实10枚有转移。其中9枚被EBUS-TBNA检测出。EBUS-TBNA的敏感性、特异性和阴性预测值分别为89%、100%和98.9%。EBUS-TBNA对早期患者的分期和指导治疗有重要意义。

局部晚期肺癌患者接受诱导化疗和放疗后是否出现降级决定了下一步治疗方案，但是治疗后再分期很困难，Kunst等^[22]^探索了TBNA再分期的价值。14例IIIa-N2 NSCLC患者接受了诱导化疗和放疗，手术前进行TBNA，共穿刺了17枚淋巴结。结果显示17枚淋巴结PET均为阳性，但5例患者是假阳性；TBNA的诊断准确性为71%。

## 4 TBNA对肺部其它疾病的诊断

首先，TBNA可用于粘膜下病变的诊断，Shure等^[23]^检查了31例这类患者，钳夹阳性率为55%，TBNA为71%，两者联合为89%。Dasgupta等^[24]^诊断55例粘膜下病变，常规支气管镜诊断阳性率为42/55（76%），而结合TBNA诊断阳性率提高至53/55（96%）。

其次，TBNA也可有效地用于结节病的诊断。日本Oki等^[25]^前瞻性研究了TBNA在结节病中的诊断意义，共15例疑诊结节病患者，先用EBUS-TBNA方法用22 G穿刺针穿刺，然后采用常规TBNA方法用19 G穿刺针穿刺。结果93%（14/15）的患者EBUS-TBNA/TBNA病理可发现非坏死性肉芽肿，结合临床表现确诊为结节病；另1例EBUS-TBNA/TBNA结果阴性者手术活检病理为恶性黑色素瘤。EBUS-TBNA共穿刺了14例结节病患者中的23枚淋巴结，其中18枚（78%）淋巴结病理阳性，13例患者得到了病理诊断。TBNA也诊断了13/14（93%）患者。Garwood等^[26]^收集了接受EBUS-TBNA的50例疑似结节病患者的临床资料，共穿刺了82枚淋巴结。通过随访48例诊断为结节病，41例（85%）患者EBUS-TBNA病理中可见非坏死性肉芽肿。Wong等^[27]^对65例疑似结节病的患者进行EBUS-TBNA检查，共穿刺了77枚淋巴结。最终61例确诊为结节病，其它4例为韦氏肉芽肿1例和未确定3例。61例结节病中56例（91.8%）EBUS-TBNA结果中可见非坏死性肉芽肿。

Tremblay等^[28]^对50例结节病患者的随机对照试验也得出了同样的结论。该研究把50例患者随机分为EBUSTBNA组和常规TBNA 19 G穿刺针穿刺组。其中，EBUSTBNA组24例患者，常规TBNA 19 G穿刺针穿刺组26例。通过双盲法进行病理诊断，常规TBNA组的诊断率、敏感性、特异性分别为73.1%、60.9%、100%。而EBUSTBNA组分别为95.8%、83.3%、100%，均优于常规TBNA组。EBUS-TBNA方法较常规TBNA 19 G穿刺针穿刺方法在诊断结节病上有明显优势。

Kennedy等^[29]^分析了TBNA在淋巴瘤中的诊断价值，回顾性分析了2005年8月-2006年12月间所有因纵隔淋巴结肿大进行EBUS-TBNA的患者资料。共236例TBNA患者，其中25例疑似淋巴瘤，包括13例疑似淋巴瘤复发和12例诊断不清患者。通过半年以上的随诊，确定其中14例为良性淋巴结肿大，11例为淋巴瘤。EBUS-TBNA诊断的敏感性和特异性分别为90.9%和100%。

Nakajima等^[30]^对于支气管囊肿压迫引起气道狭窄的患者，可以采用TBNA穿刺引流解决狭窄问题。Medford等^[31]^近期的一项小样本研究也证实EBUS-TBNA对气道闭塞患者的应用价值。

因此，TBNA可用于肺癌患者的诊断和分期，也可用于多种其它肺部疾病的诊断，应在呼吸科医师中大力推广。
